# 102. Carbapenemase producing organisms in Positive Blood and urine Cultures in a large New York Health System Reveals Emergence of NDM-Harboring Organisms and Associated Morbidity and Mortality

**DOI:** 10.1093/ofid/ofae631.039

**Published:** 2025-01-29

**Authors:** Aya Haghamad, Ryan Shou, Kirby An, Jamie Lemon, Stefan Juretschko, Vincent Streva

**Affiliations:** Northwell Health, Little Neck, NY; Northwell - Long Island Jewish Medical Center, New Hyde Park, New York; Northwell - Long Island Jewish Medical Center/North Shore University Hospital, New Hyde Park, NY; Northwell Health Laboratories, Lake Success, New York; Northwell Health laboratories, Little Neck, New York; Northwell Health Laboratories, Lake Success, New York

## Abstract

**Background:**

Infections caused by organisms harboring carbapenemase genes are a significant cause of morbidity and mortality. The prevalence of carbapenemase producing organisms (CPO) has been increasing for several years, with the majority of isolates originating in healthcare settings and containing one of the five most prevalent carbapenemase genes (*bla*_KPC_, *bla*_OXA-48-like_, *bla*_NDM_, *bla*_VIM_, *bla*_IMP_). Management of patients with CPOs is particularly challenging and knowledge of the specific carbapenemase gene harbored by these organisms is imperative for proper treatment.

**Methods:**

We conducted a retrospective multicenter study of hospitalized patients with blood and urine cultures positive for a CPO from January 2021 to December 2023 (2021-2023 blood, 2023 for urine). Blood culture PCR results were utilized to identify carbapenemase genes from these specimens. For urine specimens, phenotypic carbapenemase production was assessed by a lateral flow immunoassay (NG-Test CARBA 5). Clinical outcomes including time to appropriate therapy, length of hospital stay, 30-day hospital readmission, and patient mortality were evaluated.

**Results:**

A total of 71 CPO-positive blood cultures and 50 CPO-positive urine cultures from 10 hospitals were included. *Klebsiella pneumoniae* was the predominant CPO isolated from both blood and urine samples, urinary tract infections was the most common source of CPO bacteremia. Of the carbapenemase resistance genes detected, *bla*_NDM_ comprised 39% of CPO isolated from blood and 46% of CPO isolated from urine. Median time to appropriate therapy was 3.9 hours for blood cultures and 16 hours for urine cultures. The median length of hospital stay was 20 days for blood infections and 13 days for urine infections. Thirty-day readmission rates were 16% for blood infections and 50% for urine infections, with mortality rates of 39% for blood infections and 6% for urine infections.

**Conclusion:**

Timely identification and differentiation of carbapenem resistance genes are crucial for informing adjustments in antimicrobial therapy. Despite appropriate clinical interventions patients with CPO bacteremia had elevated mortality rates, 42% in 2022 and 33% in 2023. Notably, the primary contributor to these high mortality rates was NDM, with rates reaching 54% in 2022 and 50% in 2023.

**Disclosures:**

**All Authors**: No reported disclosures

